# Study of probe-sample distance for biomedical spectra measurement

**DOI:** 10.1186/1475-925X-10-95

**Published:** 2011-11-02

**Authors:** Bowen Wang, Shuzhen Fan, Lei Li, Cong Wang

**Affiliations:** 1 Hospital of Shandong University, No. 91, Shanda Bei Road, Jinan, 250100, P. R. China; 2 School of Information Science and Engineering, Shandong University, Jinan, 250100, P. R. China

**Keywords:** biomedical spectroscopy, fiber optic probes, probe-sample distance

## Abstract

**Background:**

Fiber-based optical spectroscopy has been widely used for biomedical applications. However, the effect of probe-sample distance on the collection efficiency has not been well investigated.

**Method:**

In this paper, we presented a theoretical model to maximize the illumination and collection efficiency in designing fiber optic probes for biomedical spectra measurement. This model was in general applicable to probes with single or multiple fibers at an arbitrary incident angle. In order to demonstrate the theory, a fluorescence spectrometer was used to measure the fluorescence of human finger skin at various probe-sample distances. The fluorescence spectrum and the total fluorescence intensity were recorded.

**Results:**

The theoretical results show that for single fiber probes, contact measurement always provides the best results. While for multi-fiber probes, there is an optimal probe distance. When a 400- μm excitation fiber is used to deliver the light to the skin and another six 400- μm fibers surrounding the excitation fiber are used to collect the fluorescence signal, the experimental results show that human finger skin has very strong fluorescence between 475 nm and 700 nm under 450 nm excitation. The fluorescence intensity is heavily dependent on the probe-sample distance and there is an optimal probe distance.

**Conclusions:**

We investigated a number of probe-sample configurations and found that contact measurement could be the primary choice for single-fiber probes, but was very inefficient for multi-fiber probes. There was an optimal probe-sample distance for multi-fiber probes. By carefully choosing the probe-sample distance, the collection efficiency could be enhanced by 5-10 times. Our experiments demonstrated that the experimental results of the probe-sample distance dependence of collection efficiency in multi-fiber probes were in general agreement with our theory.

## 1. Background

Optical spectroscopy including reflectance, fluorescence and Raman spectroscopy has been used for biomedical applications, such as for cervical cancer [1,2], lung cancer [3] and skin cancer diagnosis [4]. Fiber-based probes have been widely used in biomedical spectroscopy and biomedical imaging, which provide an effective and flexible optical interface between the spectroscopic device and the samples to be measured [5-7]. The fibers have double roles in these systems: (i) delivery of illumination light to the target; and (ii) collection and delivery of signal to the spectrometer or detector. These fiber-based probes are flexible and thus can be miniaturized and put into cavities for endoscopic measurement, or inserted into microstructures such as needles. So far, fiber probes can be made with an outer diameter less than 0.5 mm [5]. The optical probe is not only limited by size, but also the illumination and collection efficiency. However, most of the probes reported in literature are lack of optimization in illumination and collection efficiency, although this is critical for low signal detection such as fluorescence and Raman spectroscopy measurement [8,9]. In this paper, we presented a theoretical model in designing fiber optic probes for biomedical applications to maximize the illumination and collection efficiency. This model is applicable to probes with single or multiple fibers at an arbitrary incident angle. We investigated a number of probe configurations and find that contact measurement for such kind of probes is very inefficient for fiber bundles. By carefully choosing the probe and sample distance, the collection efficiency can be enhanced by 5-10 fold. Experimental results are also presented to demonstrate the probe-sample distance dependence.

## 2. Methods and experiments

### 2.1 Single fiber probe

We start from a single, bare optical fiber, which can be used as light source delivery and signal collection. This is the simplest form of optical fiber based probe, but of important practical usage [8]. When light is incident onto the sample, it will be subject to specular reflection due to refractive index mismatching at the interface and diffuse reflection due to scattering. To study the collection efficiency, it can be divided into two separate processes: (1) implementation of light transport model in the tissue and (2) light coupling between the tissue and the fiber probe. Light transport in tissue has been studied [10], which can be modeled using Monte Carlo simulations [11]. We will focus on the light coupling issues between the tissue and the fiber probe.

Assuming light is illuminated onto a semi-infinite tissue (which is always the case for *in vivo *or *ex vivo *measurement), the total intensity escaping the medium surface is [8],

(1) $$I_{esc}=I_{0}R_{sp}+I_{0}\left(1-R_{sp}\right)R_{diffuse}$$

where *I*_0 _is the incident light intensity illuminated on the medium surface. *R_sp _*is the specular reflection of the tissue surface due to index mismatching. *R_diffuse _*is a dimensionless factor, called the total diffuse reflectance. For contact measurement, the signal collected by the fiber is given by,

(2) $$I_{collect}=I_{0}R_{sp}+I_{0}\left(1-R_{sp}\right)\int_{S}\int_{S}T\left(r,r^{\prime }\right)dA^{\prime }dA=I_{0}R_{sp}+I_{0}\left(1-R_{sp}\right)R_{collect}$$

where *S *is the cross section of the fiber core, *T*(*r*) is the transport factor from the fiber through the tissue to a position *r *on the surface. *dA *and *dA' *indicate the incremental aperture area for delivery and collection. For a single fiber, the collection fraction, *f*, is defined as [8],

(3) $$f=\frac{I_{collect}-I_{0}R_{sp}}{I_{esc}-I_{0}R_{sp}}=\frac{R_{collect}}{R_{diffuse}}$$

where *I_esc _*represents the light escaped from sample surface (including specular reflection). The light collected, *R_collect_*, should be split into two parts: the light that enters the optical fiber with an angle smaller than the half-angle of the acceptance angle (*R_core_*), and the light that enters the optical fiber with an angle larger than the half-acceptance angle (*R_cladding_*). *R_core _*is guided to the detector by the fiber core, and the *R_cladding _*is lost by fiber from fiber cladding. Equation (3) can be reduced as *f *= *R_core_*/*R_diffuse_*. Both *R_core _*and *R_diffuse _*can be determined numerically by Monte Carlo simulations [11]. This is a reverse problem in that the collection efficiency is determined by measuring the collected signal divided by the total signal simulated from the sample surface. In reality, particularly for probe designing, people want to design the probe so that it can collect as much signal as possible, given the signal (*R_diffuse_*) from the surface is known (or constant).

**Figure 1:**
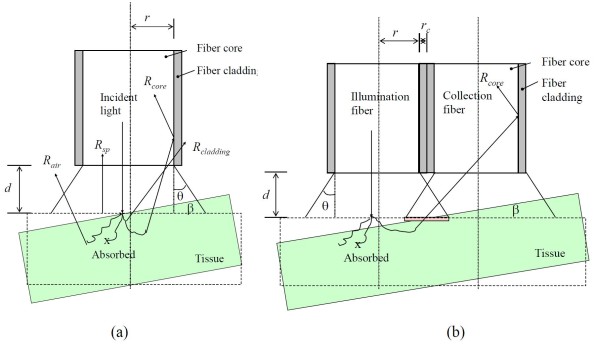
**Diagram of the possible return paths of light from tissue in a single fiber probe (a) and multi-fiber probe (b)**. *r*: radius of the fiber core, *d*: distance from the probe to the tissue surface. *R_sp_*: Specular reflectance, *R_air_*: Reflected signal to air (signal loss), *R_core_*: Collected signal that can transmit in the fiber, *R_cladding_: *Signal to the fiber cladding (signal loss), q: Acceptance angle of fiber. *r_c_*: Size of fiber cladding, β: probe tilt angle.

As shown in Figure 1, if the fiber is not contact with the tissue, the illumination and collection efficiency must be accounted for. In this case, because the energy from the fiber surface equals to the energy illuminated on the tissue surface, the intensity on the tissue surface *I*_0 _can be written as,

(4) I0=Ioutr2 cosβ∕(r+dtanθ)2

where *I_out _*is the intensity out of the fiber surface. *r *is the radius of the fiber core, *d *is the distance from the fiber tip to the tissue surface (center of fiber core to the tissue surface along optical axis of the fiber). *θ *is the acceptance angle, determined by the numerical aperture of the fiber, *θ *= arcsin(*NA*/*n*_0_). If there is no water or other medium between fiber probe and tissue surface, *n*_0 _is refractive index of air *n*_0 _= 1. NA is the numerical aperture of the fiber. For the commonly used fiber with NA = 0.22, the acceptance angle is 12.7°. *β *is the tilt angle of the fiber probe. Notice that the intensity on the sample surface maximizes for normal illumination (*β *= 0), because the illumination surface is the smallest for any given probe-sample distance. In equation (4), we assume the light is uniformly illuminated on the tissue surface.

Note that all the mirror-reflected light from the tissue surface enters the fiber. For any point on the tissue surface, only the signal which hits the fiber core surface enters the fiber, and only the signal which has a smaller incident angle than the acceptance angle can transmit in the fiber. As a first-order approximation, the solid angle, Ω, that a signal can enter the fiber core is given by,

(5a) $$\text{Ω}=1-d^{\prime }∕\sqrt{{d{}^{\prime }}^{2}+r^{2}}$$

where,

(5b) $$d^{\prime }=d+\left(r+d\tan\theta \right)\frac{\sin\beta \tan\beta \tan\theta }{\cos^{2}\beta -\sin^{2}\beta \tan^{2}\theta }$$

When the distance from the fiber probe to the tissue surface is 0, all the surface signal within the illumination area is collected, although some of the light is lost during propagation when the angle is larger than the acceptance angle. Note that when there is no tilt angle, *β *= 0, and *d' *= *d*. As the specular reflection does not provide any information about the tissue, we assume *R_sp _*= 0 for the following analysis. This is particularly useful for fluorescence and Raman measurement. Combining equations (1)-(5), one can obtain the signal that the fiber collects from the tissue surface, given by,

(6) $$R_{collect}=\frac{{\left(r+d^{\prime }\tan\theta \right)}^{2}I_{esc}}{r^{2}I_{out}}\left(1-\frac{d^{\prime }}{\sqrt{d^{{}^{\prime }2}+r^{2}}}\right)$$

In Equation (6), *I_esc _*is determined by the tissue properties that can not affected by the fiber probe. *I_out _*is the illumination intensity from the fiber output which is determined by the laser power.

**Figure 2:**
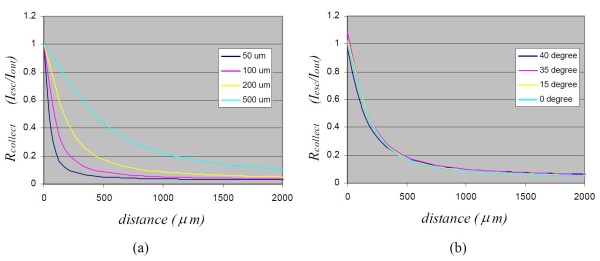
**Collection efficiency of single fiber probes with a core-diameter of 50, 100, 200 and 500 μm at normal illumination (a) and collection efficiency of single fiber probes with core-diameter of 200 μm at different illumination angles (b)**. In the above calculation, the fiber NA = 0.22 was used.

The dependence of the collection efficiency of single fiber probes on the probe distance is shown in Figure 2. It can be seen that contact measurement has the highest collection efficiency for single fiber probes. The collection efficiency also heavily depends on the diameter of the fiber core. This dependence is larger for smaller core-diameter fibers than for larger core-diameter fibers, e. g. the collection efficiency decreases to 20% of the contact measurement for a 50 μm fiber when the probe distance is 100 μm, while the collection efficiency decreases down to 20% of the contact measurement at a longer probe distance (1200 μm) for a 500 μm fiber. The collection efficiency also depends on the probe-sample angle. For any given probe-sample distance, the collection efficiency may be increased at a small tilt angle. The collection efficiency will eventually drop when the tile angle is too large. But this increase or decrease is minimal comparing with the probe-sample distance. In general, a tolerance of 15 degrees is acceptable for single-fiber probes.

### 2.2 Multi-fiber probe

For multi-fiber probes, the analysis is similar to those of single fiber probes. But because no specular reflection is collected in multi-fiber probes, *R_sp _*= 0. So the collection fraction in Eq. (3) can be rewritten as,

(7) $$f=\frac{I_{collect}}{I_{esc}}=\frac{R_{collect}}{R_{diffuse}}$$

For contact measurement, the illumination area and the collection area are not overlapped. In this case, the signal is purely diffuse reflectance signal. Based on the distance of fibers, signal at different depth in the tissue can be acquired [5]. When increasing the probe distance from the tissue surface, the illumination and collection area may be overlapped. The overlapped area over the total illumination area are given by,

(8) $$\frac{S_{collect}}{S_{illumi\;nation}}=\frac{4}{\pi }\arccos\frac{r+r_{c}}{r+d^{\prime }\tan\theta }-\frac{2\left(r+r_{c}\right)}{\pi {\left(r+d^{\prime }\tan\theta \right)}^{2}}\sqrt{{\left(r+d^{\prime }\tan\theta \right)}^{2}-{\left(r+r_{c}\right)}^{2}}$$

Combining equations (1), (4-5) and (7-8), one can obtain the signal that the fiber collects from the tissue surface in a multi-fiber probe, written as

(9) Rcollect=(r+d′tanθ)2Iescr2Iout cosβ(1-d′d′2+r2)×4 arccosr+rcr+d′tanθ(r+d′tanθ)2-2(r+rc)(r+d′ tanθ)2-(r+rc)2π(r+d′tanθ)2

In equation (9), *I_esc _*is determined by the tissue properties that can not be affected by the fiber probe. *I_out _*is the illumination intensity out of the illumination fiber, which is determined by the laser power. *r *is the radius of the fiber, *d' *is the equivalent fiber probe distance defined by equation (5b), *r_c _*is the distance between the illumination fiber and the collection fiber. *θ *is determined by the fiber NA. In the above analysis, we assume both the illumination and collection have same numerical apertures.

**Figure 3:**
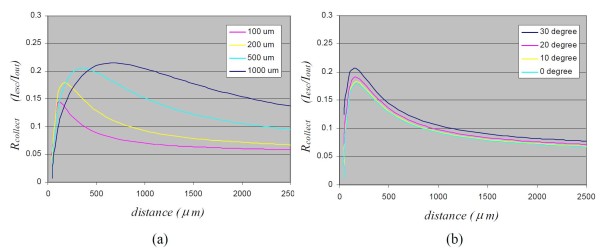
**Collection efficiency of multi-fiber probes at normal illumination-collection (a) and at different illumination-collection angles (b)**. In this simulation, the fiber NA is assumed as 0.22 NA = 0.22. *r_c _*= 10 μm. Note that there is an optimal probe distance for multi-fiber probes. This optimal probe distance depends on the diameter of fiber. The signal can be increased by 5 fold if the probe is positioned at the optimal probe distance, compared with contact measurement. In (b) the diameter of the fiber is assumed to be 200 μm.

The collection efficiency of multi-fiber probes is shown in Figure 3. It can be seen that there is an optimal probe distance in multi-fiber probes. For a 100- μm fiber, the optimal probe distance is around 20 μm; for a 1000 μm fiber, the optimal probe distance is 700 μm. Comparing with contact measurement, the improvement of multi-fibers is over 5 fold. As shown in equation (9), the collection efficiency also depends on the distance between illumination and collection fiber. In the above analysis, the distance between illumination and collection fibers is assumed to be 10 μm. Figure 3b shows the collection efficiency of multi-fiber probes at different illumination and collection angle. It can be seen that the collection efficiency is increased, because there is more overlap of illumination area and collection area for a tilted multi-fiber probes.

### 2.3 Fluorescence measurement

**Figure 4:**
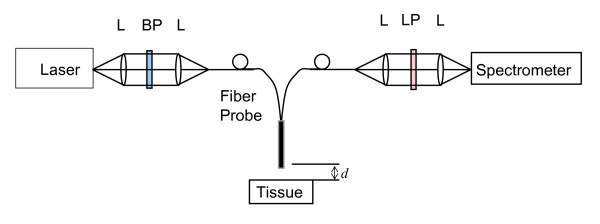
**Schematic drawing of the experimental setup**. L: lens, BP: band-pass filter, LP, long pass filter. *d*: probe distance.

In order to verify the analysis, we measured the fluorescence of a human finger skin *in vivo *using a multi-fiber probe configuration. The experiment is schematically shown in Figure 4. A laser with *λ *= 450 nm was used. The light was collimated, filtered by a band-pass filter (450 ± 1 nm) and coupled into a 400- μm delivery fiber. The light is delivered to the skin at a probe-sample distance of *d*. The laser power on skin is 2.5 mW, well below the ANSI standard. The fluorescence signal is collected by another six 400- μm fibers surrounding the excitation fiber. The collected fluorescence signal was collimated and filtered by a long pass filter (470 nm LP) and delivered to the spectrometer (USB2000+VIS-NIR, Ocean Optics, FL, USA). The probe distance can be adjusted so that the fluorescence at different probe distance can be recorded.

## 3. Results and discussions

**Figure 5:**
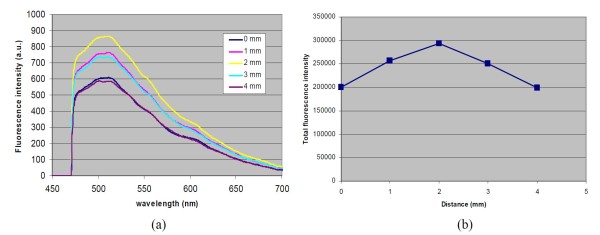
**Fluorescence of a finger skin tissue at various probe distance under 450 nm excitation (a), and total fluorescence at different probe-sample distance (b)**. Note that there is an optimal probe distance for fluorescence measurement for a multi-fiber probe. In this case, the optimal distance is found to be 2 mm.

Here we were using multi-fiber probes, so in this model, *R_sp _*= 0. The measured fluorescence of human finger skin and its probe-sample distance dependence are shown in Figure 5. It can be seen that human finger skin has very strong fluorescence between 475 nm and 700 nm under 450 nm excitation, probably due to a combination of keratin, collagen and elastin [12]. The fluorescence intensity is heavily dependent on the probe-sample distance. There is an optimal probe-sample distance that has the maximum collection efficiency. In our experiments, we found 2 mm is the optimal probe distance for our probe. This distance is higher than the theoretical value as shown in Figure 3(a). We believe that the difference between theory and experiment might be due to the multiple scatterings of the excitation light and fluorescence signal in the skin tissue. Currently we are working on Monte Carlo model [11] to combine our theory with light transport properties in tissue to improve the prediction of optimal probe-sample distance, which will be the subject of a future publication.

## 4. Conclusions

In summary, we studied the collection efficiency of fiber probes in biomedical spectroscopy and biomedical imaging. It was found that for single fiber probes, contact measurement always provides the best results. While for multi-fiber probes, there is an optimal probe distance. This optimal distance depends on the diameter of the fiber, and the distance between illumination and collection fibers. Tilted probes may also increase the collection efficiency but not as much as probe-distance effect. For normal illumination and collection, signals can be improved by 5 fold at the optimal distance than contact measurement.

## Competing interests

The authors declare that they have no competing interests.

## Authors' contributions

BW contributed in the theoretical model, proposal of the method, and writing of the manuscript. SF, LL, CW contributed equally in the experiment. All authors read and approved the final manuscript.
